# Supplementary material for: The TLK1–MK5 Axis Regulates Motility, Invasion, and Metastasis of Prostate Cancer Cells
Source: Cancers (Basel). 2022 Nov 22;14(23):5728. doi: 10.3390/cancers14235728 (PMC9736944; doi:10.3390/cancers14235728)
Supplement: Supplementary file 1 [file cancers-14-05728-s001.zip › cancers-1991888-supplementary.pdf]

cancers

The TLK1–MK5 Axis Regulates Motility, Invasion, and Metastasis of Prostate Cancer Cells

Supplementary Figure 1

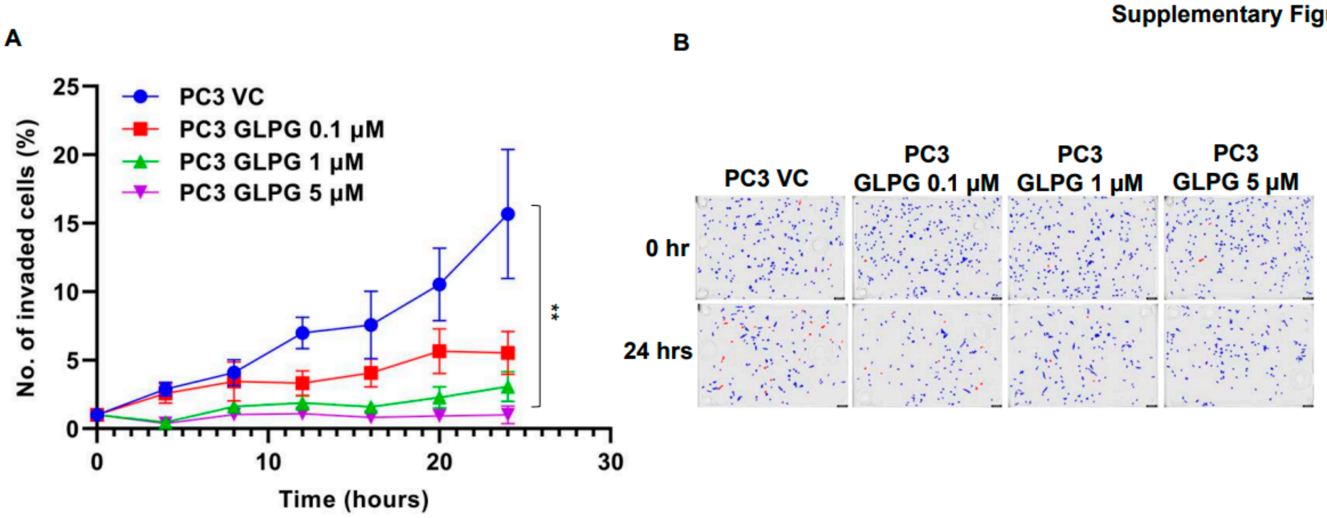

Supplementary Figure S1: MK5 inhibition impairs invasive capacity of PC3 cells. (**A**) Trans-well invasion assay was conducted to determine cellular invasion capabilities of PC3 cell treated with different concentrations of GLPG, at different time points, using Matrigel coating. The invasion rate was determined by plotting total object phase area against time; n = 5–7 biological replicates were used for each cell line. (**B**) Image representation of the trans-well invasion assay. Blue color represents the cells in the top chamber, and red color represents the cells that invaded into the bottom chamber. One-way ANOVA, followed by Tukey's post hoc analysis, was used for multiple group comparison; ** = $p < 0.005$. Error bar represents standard error of the mean (SEM). VC = vehicle control (DMSO).

Supplementary Figure 2

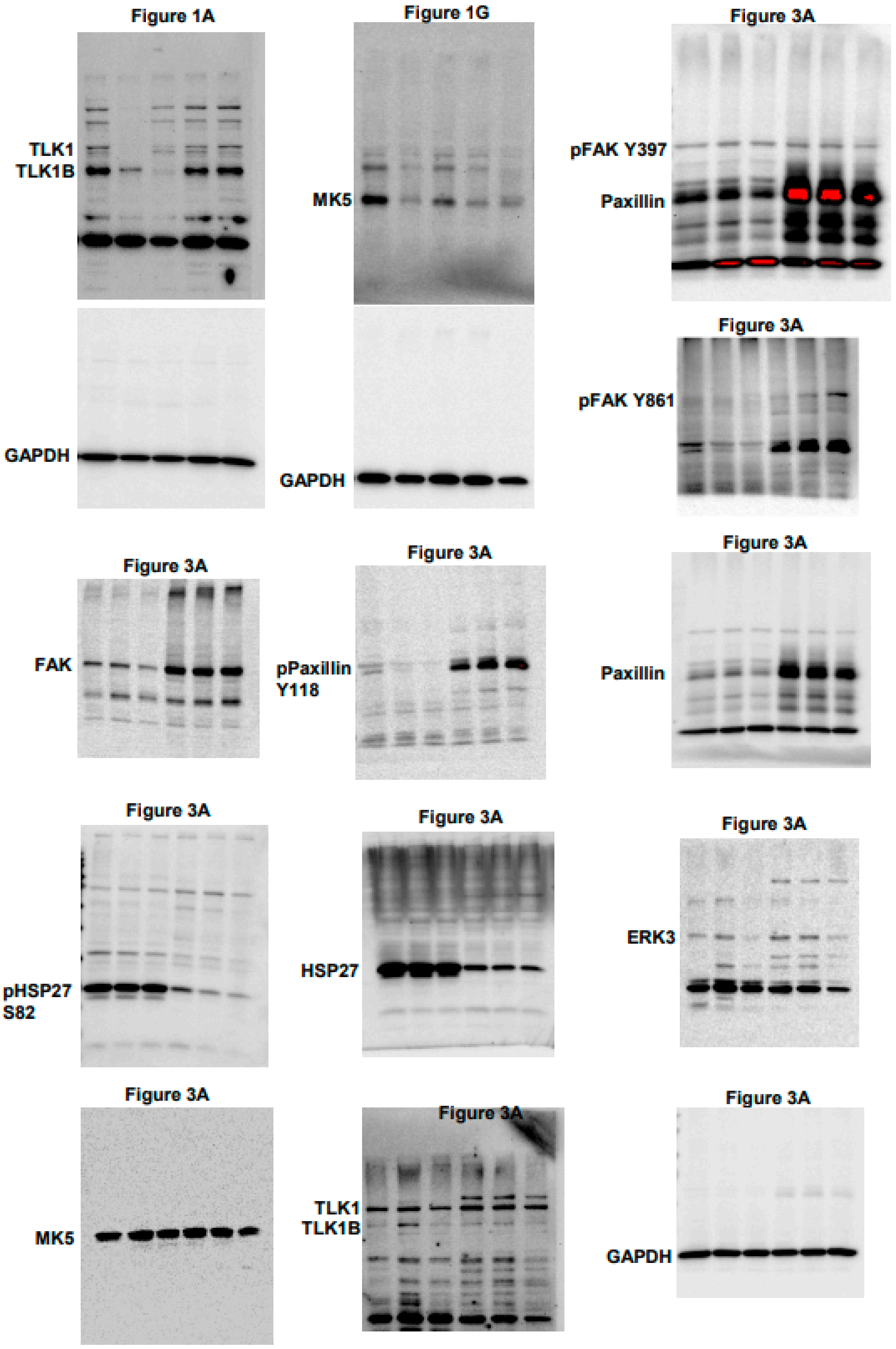

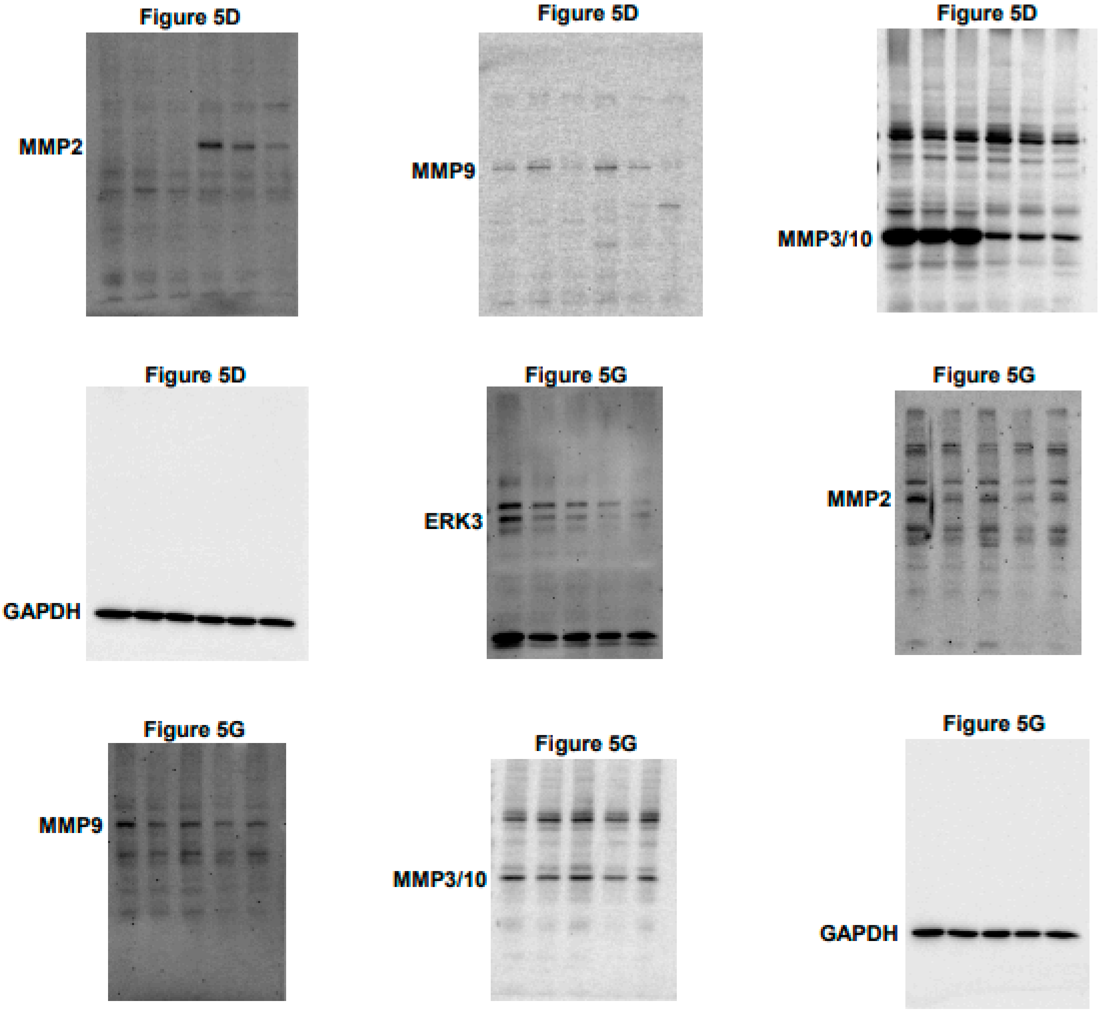

Supplementary Figure S2. Whole immunoblots of main Figures 1, 3, and 5.
